# Effects of *Lactobacillus curvatus* HY7601 and *Lactobacillus plantarum* KY1032 on Overweight and the Gut Microbiota in Humans: Randomized, Double-Blinded, Placebo-Controlled Clinical Trial

**DOI:** 10.3390/nu14122484

**Published:** 2022-06-15

**Authors:** Sung-Joon Mo, Kippeum Lee, Hyoung-Ju Hong, Dong-Ki Hong, Seung-Hee Jung, Soo-Dong Park, Jae-Jung Shim, Jung-Lyoul Lee

**Affiliations:** 1 R&BD Center, Hy Co., Ltd., 22, Giheungdanji-ro 24beon-gil, Giheung-gu, Yongin-si 17086, Korea; stal6000@hy.co.kr (S.-J.M.); joy4917@hanmail.net (K.L.); dkhong@hy.co.kr (D.-K.H.); jungsh@hy.co.kr (S.-H.J.); soodpark@hy.co.kr (S.-D.P.); jjshim@hy.co.kr (J.-J.S.); 2 Department of Gastroenterology, Vievis Namuh Hospital, 627, Nonhyeon-ro, Gangnam-gu, Seoul 06117, Korea; h2forever@vievisnamuh.com

**Keywords:** gut microbiota, *Lactobacillus curvatus* HY7601, *Lactobacillus plantarum* KY1032, obesity, overweight, body weight, probiotics

## Abstract

Obesity and overweight are closely related to diet, and the gut microbiota play an important role in body weight and human health. The aim of this study was to explore how *Lactobacillus curvatus* HY7601 and *Lactobacillus plantarum* KY1032 supplementation alleviate obesity by modulating the human gut microbiome. A randomized, double-blind, placebo-controlled study was conducted on 72 individuals with overweight. Over a 12-week period, probiotic groups consumed 1 × 10^10^ colony-forming units of HY7601 and KY1032, whereas the placebo group consumed the same product without probiotics. After treatment, the probiotic group displayed a reduction in body weight (*p* < 0.001), visceral fat mass (*p* < 0.025), and waist circumference (*p* < 0.007), and an increase in adiponectin (*p* < 0.046), compared with the placebo group. Additionally, HY7601 and KY1032 supplementation modulated bacterial gut microbiota characteristics and beta diversity by increasing Bifidobacteriaceae and Akkermansiaceae and decreasing Prevotellaceae and Selenomonadaceae. In summary, HY7601 and KY1032 probiotics exert anti-obesity effects by regulating the gut microbiota; hence, they have therapeutic potential for preventing or alleviating obesity and living with overweight.

## 1. Introduction

Obesity/overweight, resulting from excessive calorie intake and insufficient energy expenditure, is a chronic disease worldwide [1,2]. According to a report by the World Health Organization (WHO), there are >650 million obese adults, and ~1.9 billion people are overweight worldwide [3]. Abnormal fat accumulation is closely associated with metabolic and cardiovascular diseases, osteoarthritis, cancers, diabetes, and psychological conditions [4,5]. In clinical practice, obesity and living with overweight are typically assessed by expressing body weight as a function of height, and the most frequently used index is body mass index (BMI), calculated as weight in kilograms divided by height in meters squared [6]. However, BMI is an imprecise measurement that does not differentiate between different types of lean and fat masses [7]. Individuals living with obesity are characterized by accumulation of adipose tissue, including subcutaneous fat and visceral fat. In particular, abdominal visceral fat accumulation has the most detrimental consequences for health [5,8]. Furthermore, anthropometric measurements include waist circumference and hip circumference, and both values, coupled with visceral fat, are important predictors when assessing the risk of obesity [9,10].

In recent years, various studies have explored the relationships between the changes in the composition of the intestinal microbiome and body weight loss [11,12]. In humans, gut bacteria play a vital role in digestion and energy extraction from food through various mechanisms [13,14]. Probiotics can regulate the gut microbiota by influencing energy and lipid metabolism, and the secretory functions of adipose tissue [15,16,17]. Therefore, an understanding of the role of the gut microbiome in overweight and healthy individuals may lead to new strategies for treating obesity.

Probiotics, namely lactic acid bacteria (LAB), react with the mucosal environment of the gut to exert a physiological effect, and in vitro and in vivo studies can determine whether LAB exert anti-obesity effects [18,19,20,21,22,23]. In previous studies, *Lactobacillus curvatus* (*L. curvatus*) HY7601 and *Lactobacillus plantarum* (*L. plantarum*) KY1032 were found to improve lipid metabolism by decreasing plasma triglyceride levels in rats fed a high-fructose diet [24]. We also evaluated the anti-adipogenic effects of LAB in 3T3-L1 cell lines [25]. Moreover, *L. curvatus* HY7601 has been categorized as a new dietary ingredient (NDI) in the USA, and the Food and Drug Administration (FDA) have awarded generally recognized as safe (GRAS) status for *L. curvatus* HY7601 and *L. plantarum* KY1032. In our previous clinical studies, administration of these probiotics increased weight loss in individuals with overweight [26,27]. However, whether *L. curvatus* HY7601 and *L. plantarum* KY1032 can regulate abdominal visceral fat deposition and/or the gut microbiome in humans remains to be elucidated.

Herein, we explored the potential use of these probiotics in the treatment of obesity by conducting a clinical trial in humans. We investigated how these organisms influence overweight by modulating the gut microbiome and microbial diversity. Finally, we speculate on the role of the gut microbiota in the intestinal environment and obesity following supplementation with *L. curvatus* HY7601 and *L. plantarum* KY1032.

## 2. Materials and Methods

### 2.1. Test Materials

All test materials were provided by hy Co., Ltd. (Yongin, Korea). Participants were instructed to take one capsule once a day after breakfast for twelve weeks. Each 350 mg probiotic capsule contained 250 mg of *L. curvatus* HY7601 and *L. plantarum* KY1032 (5 × 10^9^ colony-forming units (cfu) each), 5.57 mg of crystalline cellulose, 3.5 mg of SiO_2_, and 7 mg of magnesium stearate. Each 350 mg placebo capsule contained 250 mg of lactose, 5.57 mg of crystalline cellulose, 3.5 mg of SiO_2_, and 7 mg of magnesium stearate. The placebo capsules looked and tasted identical to the probiotic capsules, and had the same energy content.

### 2.2. Study Subjects and Inclusion/Exclusion Criteria

The study subjects were recruited from Vievis Namuh Hospital, Seoul, Korea. A total of 72 otherwise healthy obese and overweight male and female subjects were enrolled in the study. The subjects were >19 and ≤65 years old, and had BMIs of ≥23.0 kg/m^2^ and <35.0 kg/m^2^. All subjects agreed to participate, and only those not diagnosed with any disease were included in the study. Exclusion criteria included the following: uncontrolled hypertension > 160/100 mmHg; fasting blood sugar ≥ 126 mg/dL; random blood sugar ≥ 200 mg/dL; diabetics taking antidiabetic drugs (oral hypoglycemic agents or insulin, etc.); currently being treated for serious cardiovascular, immune, respiratory, hepatobiliary, renal and urinary, nervous, musculoskeletal, psychiatric, infectious, and malignant disorders; clinically clear risk factors for gastrointestinal bleeding; diagnosed with or treated for cancer within the previous 5 years; consumed drugs affecting body weight within the last month (inhibitors and appetite suppressants); consumed functional foods/supplements for obesity improvement; received antibiotics or immunosuppressive drugs within the previous 2 weeks; lost >10% of body weight in the previous 3 months; joined any commercial obesity program within the previous 3 months; <0.1 μlU/mL or >10 μlU/mL of thyroid-stimulating hormone (TSH); creatinine levels more than twice the normal range; aspartate aminotransferase (AST) or alkaline phosphatase (ALT) three times the normal range; mental disorders, such as depression, schizophrenia, alcoholism, and drug addiction; unable to exercise due to musculoskeletal disorders; pregnant or planning to become pregnant or nursing mothers; participated in or planned to participate in other clinical trials within the previous month; sensitive or allergic to the food ingredients of the human application test; and any person deemed inappropriate by the investigator for other reasons.

### 2.3. Study Design

This 12-week randomized, double-blind, placebo-controlled study on overweight and obese Koreans was approved by the Ethics Board Committee of Vievis Namuh Hospital (IRB No. VNIRB-202108). Prescreening was conducted by a telephone conversation and subjects who met the eligibility criteria were scheduled for a baseline visit. Participants were satisfied with the inclusion and exclusion criteria. Written informed consent was obtained from all participants before enrollment. Assessments were conducted every 6 weeks, i.e., week 6 and week 12 after randomization (first visit for screening, 2 weeks; second visit, 0 weeks; third visit, 6 weeks; fourth visit, 12 weeks). At the baseline visit (second visit), subjects were randomly assigned to receive either probiotics or placebo. Randomization lists were computer-generated by a statistician. Subjects, as well as investigators, were blind to the intervention assignment until the end of the study. The required number of subjects was determined using a power calculation according to the published guidelines for human dietary intervention studies. Probiotics and placebo capsules were provided by the investigators every 6 weeks, and compliance was assessed at every follow-up. Compliance was monitored by a trained researcher by calculating the number of remaining capsules collected from participants at the third and fourth visits. Subjects were instructed to maintain their eating habits and physical activity during the study period (pre-ingestion, 2 weeks; ingestion period, 12 weeks) to ensure that any body weight fluctuations were not due to diet or physical activity. Before and after intervention, both groups were evaluated for various parameters (anthropometric variables, biochemical assessments, vital signs, energy intake, and exercise). Participants were also examined for any adverse effects during the intervention.

### 2.4. Outcomes

The primary outcomes were changes in anthropometric variables, including body weight, BMI, waist circumference and hip circumference at week 12 compared with baseline. On each visit, anthropometric variables were measured and recorded by a trained workforce. Body weight and BMI were measured by an Inbody 770 instrument (Biospace, Seoul, Korea). Waist circumference was measured directly on the skin at the umbilical level after normal expiration with the subject in an upright standing posture using a plastic measuring tape with measurements to the nearest 0.1 cm.

Secondary outcomes included body composition (body fat percentage, body fat mass, body lean mass), visceral fat area, lipid profile including total cholesterol (TC), triglyceride (TG), high-density lipoprotein cholesterol (HDL-C), low-density lipoprotein cholesterol (LDL-C), insulin, leptin, adiponectin, and high-sensitivity C-reactive protein (hs-CRP). Bioelectrical impedance analysis (BIA) was performed using an Inbody 770 instrument (Biospace) to measure body composition and visceral fat area. To assess lipid profiles, insulin, leptin, adiponectin, and hs-CRP, blood samples were collected after 12 h overnight fasting. Serum concentrations of lipid profiles, including TG, TC, LDL-C, and HDL-C, were measured by colorimetric methods using appropriate commercial kits. Serum insulin, leptin, adiponectin and hs-CRP concentrations were measured by enzyme-linked immunosorbent assay using dedicated kits.

### 2.5. Safety

For all subjects, before and after 12 weeks of intervention, vital signs (blood pressure, pulse rate) and biochemical parameters were assessed. Diastolic blood pressure (DBP) and systolic blood pressure (SBP) were assessed in the supine position after a resting period (20 min). Vital signs were measured twice on the left arm with an automatic BP monitor, and the average of the two measurements was used. For all subjects, before and after 12 weeks of intervention, biochemical parameters were evaluated using a Siemens ADVIA 1800 instrument (Siemens, München, Germany), including white blood cells, red blood cells, hemoglobin, hematocrit, platelets, lymphocytes, aspartate aminotransferase, alanine aminotransferase, total bilirubin, gamma-glutamyl transpeptidase, creatinine, blood urea nitrogen, and uric acid in whole blood, serum, plasma, and urine.

### 2.6. Fecal Microbiome Analysis

#### 2.6.1. Sample Handling and Collection

Stool samples were collected at two study time points; prior to intervention (zero weeks) and at twelve weeks. Sample collection was performed with fecal collection tubes, and samples were transported to the laboratory on ice bags, after which they were frozen and stored at −80 °C until use. DNA was isolated in multiple batches to reach the desired quantity using a MoBio Power Soil DNA Isolation Kit (Qiagen, Hilden, Germany) according to the manufacturer’s instructions. DNA samples were carefully quantified with a Nanodrop spectrophotometer (Thermo Scientific, Waltham, MA, USA), and A260/A280 ratios were measured to confirm the high-purity DNA yield. DNA samples were frozen and stored at −20 °C until use.

#### 2.6.2. 16S rRNA Gene Amplicon Sequencing

Sequencing libraries were prepared according to Illumina 16S Metagenomic Sequencing Library protocols to amplify the V3 and V4 regions. The input gDNA (2 ng) was PCR-amplified with 5× reaction buffer, 1 mM dNTP mix, 500 nM of each universal F/R PCR primer, and Herculase II fusion DNA polymerase (Agilent Technologies, Santa Clara, CA, USA). Thermal cycling for the first PCR step included a 3 min denaturation at 95 °C, 25 cycles of 30 s at 95 °C, 30 s at 55 °C, and 30 s at 72 °C, followed by a 5 min final extension at 72 °C. The universal primer pair with Illumina adapter overhang sequences used for the first amplifications were V3-F (5′-TCGTCGGCAGCGTCAGATGTGTATAAGAGACAGCCTACGGGNGGCWGCAG-3′) and V4-R (5′-GTCTCGTGGGCTCGGAGATGTGTATAAGAGACAGGACTACHVGGGTATCTAATCC-3′). The PCR product from the first step was purified using AMPure beads (Agencourt Bioscience, Beverly, MA, USA). Following purification, 2 μL of PCR product from the first step was PCR amplified for final library construction using NexteraXT Indexed Primer (Illumina, San Diego, CA, USA). Thermal cycling of the second PCR step was performed as described for the first step, but with 10 cycles instead of 25 cycles. The resulting PCR product was purified with AMPure beads, and quantified using quantitative PCR (qPCR) according to the qPCR Quantification Protocol Guide (KAPA Library Quantification kits for Illumina Sequencing platforms) and qualified using a TapeStation D1000 ScreenTape (Agilent Technologies, Waldbronn, Germany).

#### 2.6.3. Analysis of Operational Taxonomic Units (OTUs)

After sequencing, Illumina MiSeq raw data were sorted by sample using index sequences, and paired-end FASTQ files were generated for each sample. The sequencing adapter sequence and F/R primer sequence of the target gene region were removed using Cutadapt (v3.2, https://cutadapt.readthedocs.io/en/stable/, accessed on 5 April 2022) [28]. To correct errors in the amplicon sequencing process, the DADA2 (v1.18.0, Nashville, USA) package of the R (v4.0.3, The R Foundation, Indianapolis, IN, USA) program was used [29]. In the case of paired-end reads, the forward sequence (Read1) and reverse sequence (Read2) were cut into 250 bp and 200 bp fragments, respectively, and sequences with an expected error of two or more were excluded. An error model was then set up for each batch and noise was removed from each sample. After sequencing, error-corrected paired-end sequences were assembled into one sequence, and chimeric sequences were removed using the DADA2 consensus method, resulting in OTUs.

In addition, for comparative analysis of the microbial community, the QIIME (v1.9, J Gregory Caporaso, Fort Collins, CO, USA) program was used to apply and normalize subsampling based on the number of reads in the sample using the minimum number of reads from the total sample [30]. BLAST+ (v2.9.0, Bethesda, Rockville, MD, USA) was used with the Reference database (NCBI 16S Microbial DB) for each OTU sequence, and classification information for the organism with the highest similarity was acquired [31]. If the query coverage of the best hit that matched the DB was <85%, or the identity of the matched area was <85%, taxonomy information was not acquired. In addition, for multiple alignments between OTU sequences, the mafft (v7.475, https://mafft.cbrc.jp/alignment/software/, accessed on 5 April 2022) program and the FastTreeMP (v2.1.10, http://www.microbesonline.org/fasttree/, accessed on 5 April 2022) program were used to generate phylogenetic trees [32,33].

Comparative analyses of different microbial communities were performed using QIIME, in conjunction with the above OTU abundance and classification information. The observed species, ACE index, and Shannon index were obtained to confirm the species diversity and uniformity of the microbial community in the sample, and alpha diversity information was confirmed by the Chao1 index. Based on weighted and unweighted UniFrac distances, beta diversity between samples (information about microbial community diversity between samples in comparison groups) was determined, and relationships between the samples were visualized using principal coordinate analysis (PCoA) plots. Linear discriminant analysis effect size (LEfSe) was performed using LEfSe software (v1.0, https://huttenhower.sph.harvard.edu/galaxy/, accessed on 15 April 2022).

### 2.7. Statistics

Statistical analysis was performed with the SAS version 9.4 software package (SAS Institute, Cary, NC, USA). For continuous variables, normality tests were performed using Shapiro–Wilk tests. Variables that were normally distributed were tested for significance using two-sample *t*-tests, and variables that were not normally distributed were tested for significance using Mann–Whitney U-tests. Within-group comparisons were assessed using paired *t*-tests. In addition, changes from the baseline in each parameter between the two groups were analyzed using analysis of covariance (ANCOVA) adjusted for baseline values. Pearson correlation analysis using R (www.R-project.org, accessed on 25 April 2022) was performed to evaluate the relationship between the relative abundance of the gut microbiota and the corresponding anthropometric parameters (body weight, BMI, waist circumference, body fat mass, and visceral fat area), with minimal abundance > 0.5%. The results are expressed as the mean ± standard error of means (SEM), and *p* < 0.05 was considered statistically significant.

## 3. Results

### 3.1. Baseline Characteristics of Subjects

**Figure 1:**
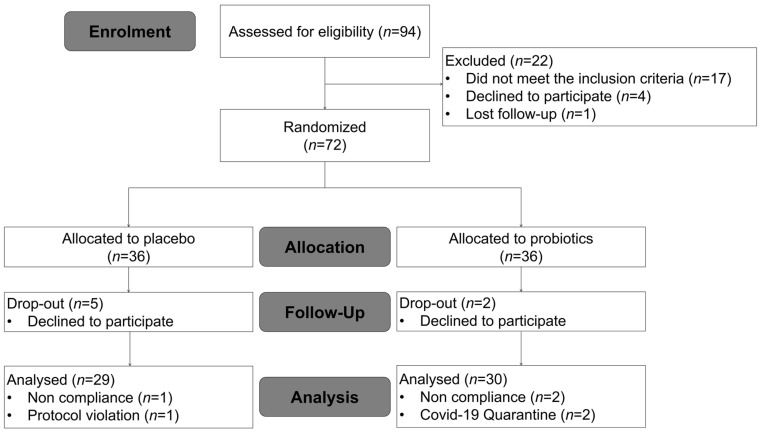
Flow chart illustrating the steps for screening, enrollment, assignment, and follow-up of study participants for per protocol (PP) analysis.

Ninety-four subjects were recruited, and seventy-two eligible subjects were enrolled and randomized into two groups. Eight subjects were withdrawn during the intervention period (five in the placebo group and three in the probiotics group) because they declined to participate. A total of sixty-four subjects completed the study; however, three subjects with <70% treatment compliance, one subject who did not follow habitual physical activity patterns, and two participants who self-quarantined due to COVID-19 infection during the study were excluded from the analysis. Therefore, 30 subjects in the probiotics group and 29 subjects in the placebo group were included in the data analysis (Figure 1). No adverse events were reported as a dropout reason. Among the subjects completing the 12 weeks of treatment, medication compliance was 98.64% and 96.48% in the treatment and placebo groups, respectively (*p* = 0.476). The baseline characteristics of the subjects are listed in Table 1. The two groups were well matched for age, gender distribution, smoking, and drinking. There were no significant differences between the two groups in anthropometric variables, vital signs, lipid profiles, insulin, leptin, adiponectin, or hs-CRP.

### 3.2. Efficacy Analysis

#### 3.2.1. Anthropometric Measurement of Body Composition and Visceral Fat Area

**Figure 2:**
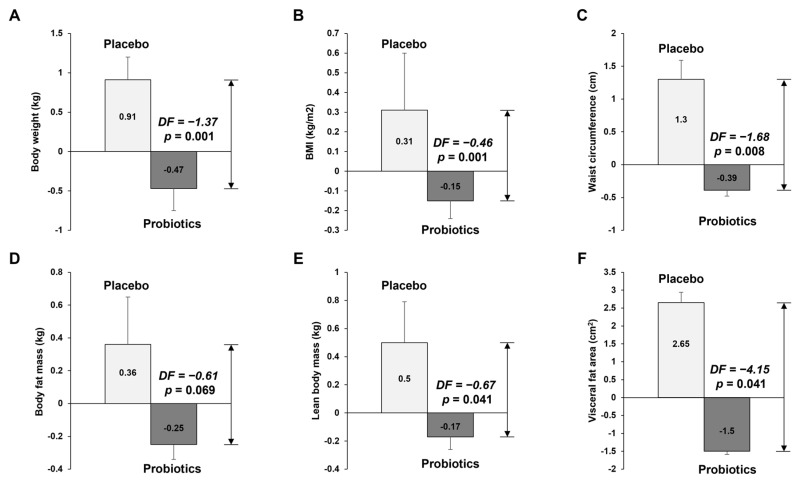
Results of anthropometric variable measurements. The graphs show (**A**) body weight, (**B**) BMI, (**C**) waist circumference, (**D**) body fat mass, (**E**) lean mass, and (**F**) visceral fat area, which all decreased in the probiotics group after 12 weeks of intervention. The data points correspond to the mean ± SEM. *p*-value are derived from ANCOVA tests adjusted for baseline values. DF, difference of least square mean from placebo group; LS mean, least square mean.

Table 2 and Figure 2 show the anthropometric parameters and body composition at baseline and 12 weeks for placebo and probiotic groups. After 12 weeks of probiotic intake, the mean values of body weight, BMI, and waist circumference decreased compared with baseline values, but those in the placebo group increased compared with the baseline values. We compared the anthropometric and body composition changes (differences from the baseline) between the placebo and probiotic groups. The probiotic group had greater reductions in body weight (Δ0.93 kg vs. Δ−0.47 kg, *p* = 0.001), BMI (Δ0.32 kg/m^2^ vs. Δ−0.15 kg/m^2^, *p* < 0.001), waist circumference (Δ1.31 cm vs. Δ−0.41 cm, *p* = 0.007), body fat mass (Δ0.39 kg vs. Δ−0.28 kg, *p* = 0.043), lean body mass (Δ0.51 kg vs. Δ−0.18 kg, *p* = 0.032), and visceral fat area (Δ2.82 cm^2^ vs. Δ−1.67 cm^2^, *p* = 0.025). At 12 weeks, there were no significant differences between the two groups for any variables (*p* > 0.05 by independent *t*-test). However, differences in body weight (79.99 kg vs. 78.74 kg, *p* = 0.001), BMI (27.13 kg/m^2^ vs. 26.73 kg/m^2^), waist circumference (96.23 cm vs. 93.58 cm, *p* = 0.008), lean body mass (55.92 kg vs. 57.16 kg, *p* = 0.041), and visceral fat area (2.82 cm^2^ vs. −1.67 cm^2^, *p* = 0.041) between the two groups at 12 weeks were statistically significant, as analyzed by independent *t*-tests adjusted for baseline values.

#### 3.2.2. Measurement of Biochemical Variables in Blood

**Figure 3:**
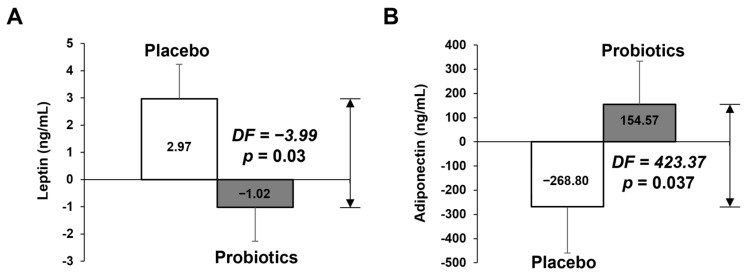
Adipokines measured via ELISA at baseline and at 12-weeks’ follow-up and mean changes according to treatment. (**A**) Leptin, (**B**) adiponectin. The data points correspond to the mean ± SEM. *p*-value are derived from ANCOVA tests adjusted for baseline values. DF, difference of least square mean from placebo group; LS mean, least square mean.

Table 3 and Figure 3 shows blood lipid profile, insulin, leptin, adiponectin, and hs-CRP parameters at baseline and at week 12. The change in adiponectin in the probiotics group was significantly different that than of the placebo group after 12 weeks (−246.83 ng/mL vs. 133.33 ng/mL, *p* = 0.046), but did not in leptin. However, the difference in change of leptin between the two groups was statistically significant (2.97 ng/mL vs. −1.02 ng/mL, *p* = 0.03), as analyzed by ANCOVA tests adjusted for the baseline values (Figure 3A). Adiponectin also showed a significant difference in the ANCOVA test (Figure 3B).

### 3.3. Microbiome Analyses

#### 3.3.1. Composition of the Gut Microbiota, and Shifts in Bacterial Alpha and Beta Diversity

**Figure 4:**
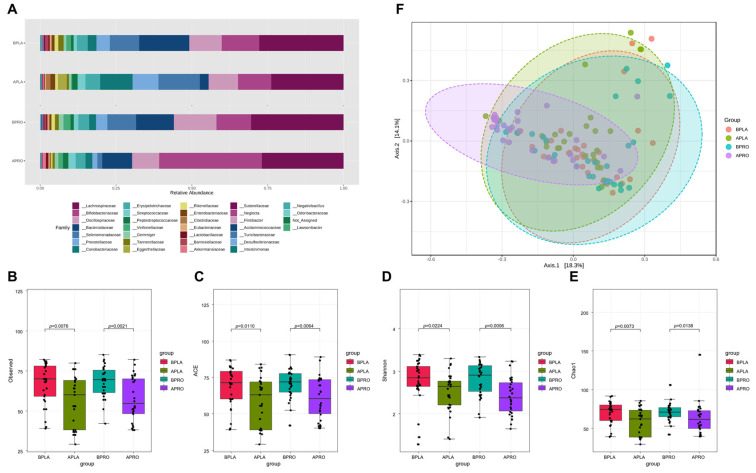
Bacterial family abundance, alpha diversity, and beta diversity. (**A**) Relative abundance at the family level at baseline and after intervention in placebo and probiotic groups. Boxplots show the alpha diversity of bacterial communities at baseline and after intervention in placebo and probiotics groups for (**B**) observations and (**C**) ACE, (**D**) Shannon, and (**E**) Chao1 indices. (**F**) Principal coordinate analysis (PCoA) showing the microbial community distance between baseline in the placebo group (orange circles), after intervention in the placebo group (green circles), baseline in the probiotic group (blue circles), and after intervention in the probiotic group (purple circles). Each group is identified by a different color, as shown to the right of the figure. BPLA, before placebo; APLA, after placebo; BPRO, before probiotics; APRO, after probiotics.

Taxonomic differences were observed between groups (Figure 4). At the family level, Bifidobateriaceae and Akkermansiaceae increased, while Oscillospiraceae-, Selenomonadaceae, and Prevotellaceae decreased in the probiotics group after intervention. After placebo intervention, Selenomonadaceae, Prevotellaceae, Coriobacteriaceae, and Eggerthellaceae increased and Bacteroidaceae decreased.

Fecal microbiome community alpha diversity in the four groups (baseline placebo, after placebo, baseline probiotics and after probiotics) was characterized using observed species, Shannon index, ACE index, and Chao1 index. Sequencing data and alpha diversity index for each sample are presented in Figure 4B–E. Significant differences in community richness were observed across all groups. Richness was lowest in the placebo group after intervention, and was significantly different from the baseline. Likewise, baseline alpha diversity of the probiotics group had higher richness than after intervention. Furthermore, differences were statistically significant compared with the baseline and after intervention in each group. The microbiota community structure of each group was examined by PCoA, and the probiotics group after intervention had the most variability, whereas the placebo group showed the lowest variability.

#### 3.3.2. LEfSe

**Figure 5:**
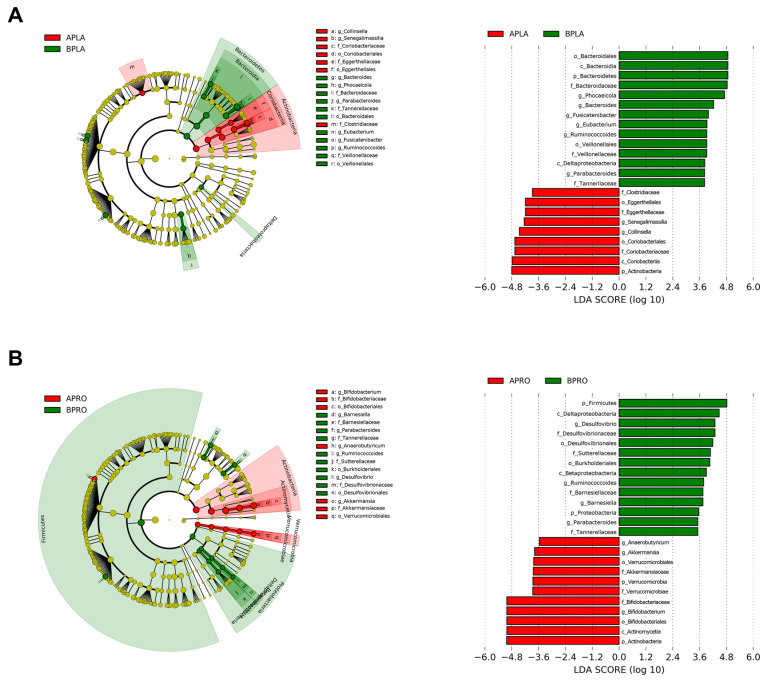
Linear discriminant analysis (LDA) effect size (LEfSe) cladogram and plots of microbial taxa between baseline and after intervention for placebo (**A**) and probiotics (**B**) group. The LEfSe algorithm was applied using the Galaxy computational tool v.1.0. (https://huttenhower.sph.harvard.edu/galaxy/, accessed on 15 April 2022). The diameter of each circle is proportional to the abundance of the taxon. The length of the bar represents the log10 transformed LDA score. The threshold on the logarithmic LDA score for discriminative features was set to 3.0. Only taxa of bacteria with statistically significant changes (*p* < 0.05) in relative abundance is written on the horizontal line. The name of the taxon level is abbreviated as p—phylum; c—class; o—order; f—family, and g—genus. BPLA, before placebo; APLA, after placebo; BPRO, before probiotics; APRO, after probiotics.

LEfSe was applied to identify bacteria with significantly increased or decreased relative abundance between baseline and post-intervention. A circular cladogram and plots show the differentially abundant taxa between baseline and post-intervention in each group (Figure 5). In the placebo group, relative abundance after intervention was significantly higher for members of phylum Actinobacteria (e.g., the class Coriobacteriia; the family Coriobacteriaceae and Eggerthellaceae; the genus *Collinsella* and *Senegalimassilia*), and significantly lower for members of phylum Bacteroidetes (e.g., the family Tannerellaceae and Bacteroidaceae; the genus *Bacteroides*, *Phocaeicola*, and *Parabacteroides*), compared to the baseline (Figure 5A). In the probiotics group, relative abundance after intervention significantly increased for members of phylum Actinobacteria (e.g., the genus *Bifidobacterium*), as well as Verrucomicrobia (e.g., the genus *Akkermansia*). In addition, relative abundance after intervention significantly decreased for members of phylum Firmicutes (e.g., the genus *Ruminococcoides*) and Proteobacteria (e.g., the family Sutterellaceae and Desulfovibrionaceae; the genus *Desulfovibrio*) (Figure 5B).

#### 3.3.3. Comparative Analysis of the Gut Microbiota at the Species Level

**Figure 6:**
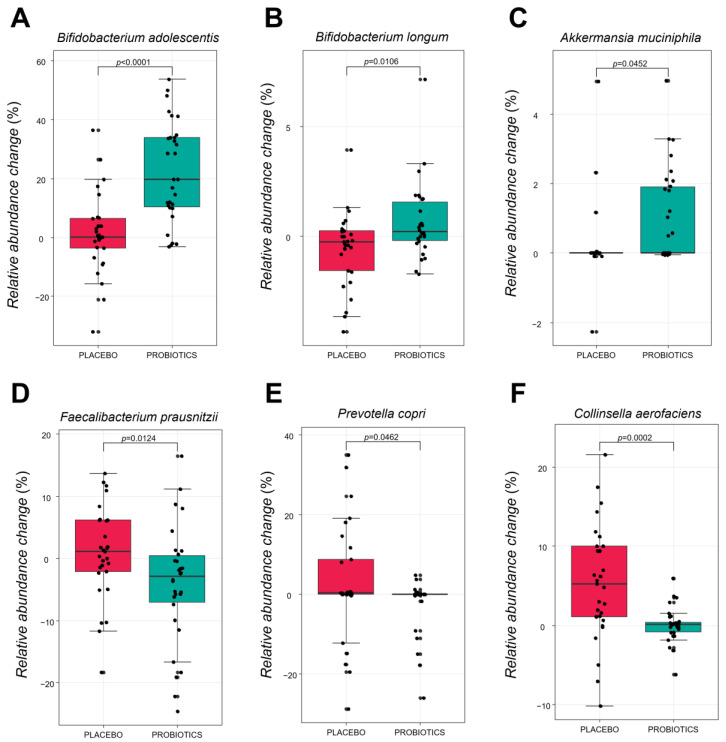
Relative abundance changes of six fecal microbiota species at baseline and after intervention in each group. (**A**) *Bifidobacterium adolescentis*. (**B**) *Bifidobacterium longum*. (**C**) *Akkermansia muciniphila*. (**D**) *Faecalibacterium prausnitzii*. (**E**) *Prevotella copri*. (**F**) *Collinsella aerofaciens*. Species are shown in the boxplot. The *p*-values were obtained by performing Mann–Whitney U-tests for differences between groups at baseline or after intervention.

Figure 6 shows the specific bacterial species that changed after intervention in the placebo and probiotic groups. There was a statistically significant increase in the relative abundance change value of *B. adolescentis*, *B. longum* and *A.muciniphila* in the probiotic group (*p* < 0.0001, *p* = 0.0106, and *p* = 0.0452, respectively), compared with placebo group. By contrast, the relative abundance change value of *Collinsella aerofaciens*, *Faecalibacterium prausnitzii*, and *Prevotella copri* significantly decreased compared to the placebo group (*p* = 0.0002, *p* = 0.0124, *p* = 0.0462, respectively). It implies that supplementation of HY7601 and KY1032 for 12 weeks could regulate intestinal microbial species in individuals with overweight.

#### 3.3.4. Pearson Correlation Analysis

*B. bifidum* and *B. adolescentis* of the genus Bifidobacterium, and *A. muciniphila* of the genus Akkermansia, were found to be abundant in feces after the ingestion of probiotics, and were negatively correlated with body measurements such as body weight, BMI, waist circumference, body fat mass, and visceral fat area. By contrast, after 12 weeks of placebo intervention, the abundances of *Prevotella copri* and *Megamonas funiformis* were positively correlated with anthropometric parameters.

## 4. Discussion

Probiotics are defined by the WHO as live microorganisms that, when administered in adequate amounts, confer a health benefit on the host [34]. Most bacteria with probiotic properties belong to the genus *Lactobacillus* and *Bifidobacterium*, which are common but non-dominant members of the indigenous microbiota of the human gastrointestinal system [35,36]. Probiotics can be considered functional foods, due to the health benefits they confer outside of their traditional nutritional functions [37,38]. In particular, recent studies suggest that probiotics play a role as natural therapeutic supplements with the potential to improve lipid metabolism. Furthermore, recent studies have suggested the potential for weight control through interventions that affect gut microbiome diversity [15,39].

According to a previous study, the alpha diversity in mice fed a high-fat diet with HY7601 and KY1032 isolated from Korean fermented kimchi at 10^10^ cfu/day could significantly recover as much as those in the control group [40]. This study also reported that supplementation with probiotics could affect fat accumulation by increasing the abundance of *Lachnospiraceae*, which are known to play a role in butyrate production [41]. In a previous clinical trial, supplementation with a combination of HY7601 and KY1032 delivered to non-diabetic, overweight subjects for 12 weeks resulted in a significant reduction in plasma ox-LDL, a marker of oxidative stress [26]. However, clinical trials exploring the microbiome have not been conducted. Therefore, the purpose of the present study was to investigate whether probiotic treatment with HY7601 and KY1032 results in changes in microbial diversity and obesity.

In this study, *L. curvatus* HY7601 and *L. plantarum* KY1032 were provided daily before meals for 12 weeks, and their effects on obesity-related factors were examined in individuals with overweight. There were no significant differences in gender, age, or history of individual habits, including drinking, medicinal treatment, smoking, and medication use between the placebo and the experimental group receiving *L. curvatus* HY7601 and *L. plantarum* KY1032. Dietary intake was based on self-reports obtained from weighed food, and measurement errors from self-reported dietary intake and lifestyle variables have been reported to be relatively small. The supplementation of the diet with *L. curvatus* HY7601 and *L. plantarum* KY1032 decreased body weight, waist circumference, body fat mass, and visceral fat area in a randomized controlled human trial, but there was no significant change in body fat percentage in the present work. Probiotic-induced weight loss was associated with reductions in body fat mass measured using BIA, which was positively correlated with changes in the gut microbiome composition. These results suggest a beneficial effect of supplementation with HY7601 and KY1032 on body weight, body fat, and waist and hip circumference in overweight subjects.

An analysis of biochemical variables showed that leptin levels were significantly different between the placebo and probiotic groups. In addition, there was a significant difference in adiponectin between the placebo and probiotic groups. Leptin and adiponectin are important indicators of obesity. Leptin is known as the obesity hormone and is mainly produced by adipocytes. There is a strong correlation between leptin levels and body fat percentage, and it can be assumed that obese people are insensitive to leptin signaling [42,43]. A recent study reported that food intake, including probiotics, affects the gut microbiota composition and determines which metabolites are produced by the gut microbiota [44]. These metabolites include short-chain fatty acids and secondary bile acids, which in turn, can bind to their receptors, and thereby activate specific signaling pathways in obese humans. According to a recent study, leptin levels are high and correlate with the BMI and the percentage of body fat [45]. Furthermore, they can also modulate hormone secretion, such as leptin, which exert their effects in the brain via the circulation or by binding to the nerves. Leptin is an adipocyte hormone that regulates appetite by binding to hypothalamic receptors and causing signaling when enough food is consumed. In normal individuals, low levels of leptin cause hunger and increase food intake. However, high leptin levels in obese people suggest that they may have leptin resistance, since it become insensitive in individual with overweight [46]. Therefore, our findings suggest that decreased leptin due to probiotic 12 weeks intake effectively restored the hypothalamic leptin sensitivity. Adiponectin is a hormone secreted by adipose tissue that has an appetite suppressant effect, and is known to be lower in obese people [47]. In addition, adiponectin is known to influence body fat regulation by affecting MP-activated protein kinase (MAPK) and peroxisome proliferator-activated receptor alpha (PPARα) activities [48]. Adipokines have pleiotropic functions in the regulation of energy metabolism, as well as in appetite. In obese patients, visceral adipose tissue may affect health conditions, via an abnormal production of adipokines [49,50]. Especially, adiponectin plays a pivotal role in energy metabolism, as well as regulating BMI, glucose, insulin and triglyceride levels [51]. In various clinical trial studies, it has been reported that adiponectin concentrations decrease in individuals living with obesity and increase after weight loss [52]. Although this effect was not observed in previous clinical trials, the present trial clearly showed significant differences in leptin and adiponectin between the placebo and probiotics groups.

Over the last decade, growing evidence has revealed that the gut microbiota are a potential factor in the pathophysiology of both obesity and related metabolic disorders. In our previous clinical study, *L. curvatus* HY7601 and *L. plantarum* KY1032 were found to regulate abdominal visceral fat deposition, but their effect on the human gut microbiome was not investigated. The present study revealed differences in the gut microbiota between overweight and normal-weight individuals, as reported previously in animal studies. We examined the gut microbial composition by performing 16S rRNA gene amplicon sequencing on human stool samples and compared the results between the placebo group and the probiotics group. We aimed to investigate the effect of HY7601 and KY1032 administration on the levels of major gut microbiota in the human body, and to determine whether there is a correlation between obesity and weight loss.

We found that probiotics altered microbial composition, and that obesity and KY1032 and HY7601 administration were associated with the presence of distinct microbial taxa [53,54]. In our previous in vivo study, the relative abundance of Bifidobacterium was much higher in the HFD-probiotic mice than in the HFD-placebo mice. Indeed, Bifidobacteriaceae and Akkermansiaceae were more abundant in the probiotics group than the other three groups (Figure 4A). This implies that the increases in Akkermansiaceae and Bifidobacteriaceae observed previously are associated with body weight loss, which suggests links between these bacterial families and overweight. In contrast, the abundance of Selenomonadaceae and Prevotellaceae, which are known to have higher abundance in individuals living with obesity, was lower in the probiotics group in our study [55,56,57]. Indeed, according to the observed, ACE, Shannon, and Chao1 indices, there were significant differences in alpha diversity of the intestinal microbial community between groups (Figure 4B–E). Interestingly, the PCoA scatter plot for the probiotics group differed from the plots for the other groups (Figure 4F). This implies that KY1032 and HY7601 administration altered microbiome composition and shifted beta diversity.

In addition, changes in the gut microbial diversity associated with body weight loss were subjected to LEfSe and relative abundance analysis by grouping the study subjects into 0 week and 12 week groups. Based on the results, there was a significant enrichment in the genus Bifidobacterium and Akkermansia in the probiotics group after intervention, while the genus *Collinsella* and *Senegalimassilia* were enriched in the placebo group. Furthermore, at the species level, the change value of *B. adolescentis*, *B. longum*, and *A. muciniphila* significantly increased in the probiotics group, compared with the placebo group. Recent studies reported that obesity induces gut microbiota dysbiosis and decreases the abundance of *Bifidobacterium*. Especially, certain strains of *B. adolescentis* have been shown to alleviate obesity by modifying the gut microbiota of obese mice [58,59]. In addition, it has also been reported that a therapeutic trial focusing on *A. muciniphila* in the gut microbiota could be considered as a promising strategy for the prevention of obesity and metabolic disorder disease [60]. By contrast, changing levels of *P. copri*, *F. prausnitzii*, and *C. aerofaciens* also showed a significant difference in the probiotics compared to placebo group. According to a recent paper, the levels of *F. prausnitzii*, *P. copri*, and *C. aerofaciens* were found to be significantly higher in obese subjects than in non-obese subjects. Especially, according to other studies, the levels of *F. prausnitzii* were significantly higher in obese children group than in non-obese children. They reported that higher *F. prausnitzii* in the obese group suggests that it increases energy salvage from unabsorbed carbohydrates that would not contribute to dietary energy intake. In addition, interestingly, it has been confirmed that *F. prausnitzii* were significantly reduced in frail elderly individuals and in patients with diarrhea and malnutrition [61,62,63]. Further experiments were conducted to determine whether these regulation of intestinal microbiome was caused by ingestion of HY7601 and KY1032. (Figure S1). Acid and bile tolerance as well as survival rate in physiological conditions similar to those of the human GIT of Lactobacillus strains were evaluated, as described in studies [64,65]. As a result, HY7601 and KY1032 have showed greater acidic and bile tolerance than each Korean Collection for Type Cultures (KCTC) type strains. The HY7601 finally survived more than 35% after undergoing oral, gastric, and intestinal environments, while KY1032 survived more than 80%. Our data suggest that HY7601 and KY1032 have intestinal stability with high survival ability, which may have contributed to regulate the gut microbiome. Taken together, the administration of HY7601 and KY1032 is expected to regulate the proportion of bacteria known to contribute to or alleviate obesity.

**Figure 7:**
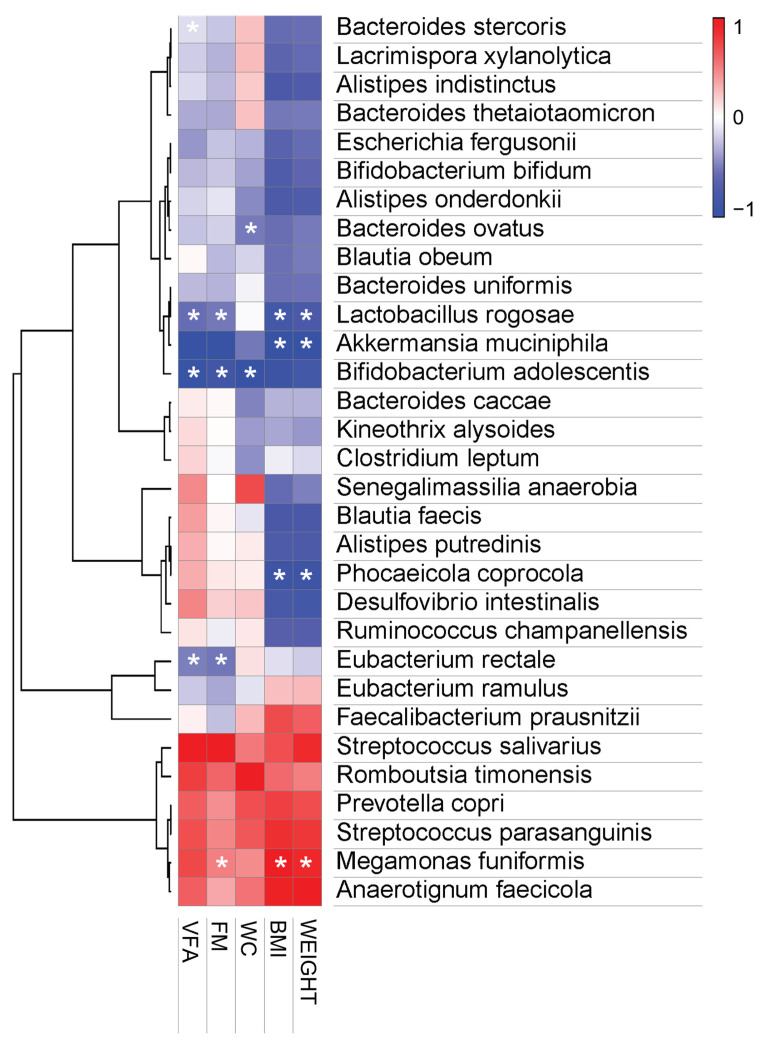
Pearson correlation analysis. Pearson’s correlation analysis was performed between the bacterial species-level taxa and the measured anthropometric values. Heatmaps were generated in MORPHEUS software (https://software.broadinstitute.org/morpheus/, accessed on 15 April 2022). In heatmaps, red squares indicate significant positive correlations (*r* > 0.3) and blue squares indicate significant negative correlations (*r* < − 0.3). (* *p* < 0.05). WC, waist circumference; FM, fat mass; VFA, visceral fat area.

Finally, we investigated the correlation between human obese parameters and the relative abundance of the microbial flora and found that *B. adolescentis* and *A. muciniphila* were negatively correlated with increases in the anthropometric variables (Figure 7). In addition, the correlation analysis indicated that body weight gain and fat mass gain were positively correlated with *M. funiformis* and *P. copri*, while *P. coprocola*, *L. rogosae*, and *Eubacterium rectale* were negatively correlated with obesity indicators (body weight, BMI, fat mass, and visceral fat area). These results indicate that there is a link between HY7601 and KY1032 administration and loss of visceral fat mass, suggesting that they have an anti-obesity effect. Additionally, these positively or negatively correlated bacterial changes can serve as important indicators of the effectiveness of HY7601 and KY1032 administration.

In conclusion, we found that HY7601 and KY1032 supplementation is a dietary intervention that could help prevent obesity and overweight. Specifically, these probiotics decreased body weight, visceral fat mass, waist circumference, and increased adiponectin. Furthermore, these probiotics changed the bacterial gut microbiota characteristics associated with each obesity indicator. HY7601 and KY1032 exerted anti-obesity effects by regulating the gut microbiota composition, which could lead to effective therapeutic trials. The findings demonstrate that HY7601 and KY1032 intake can alter the composition and diversity of the human gut microbiome, and thereby help prevent obesity and its associated metabolic syndrome.

## Figures and Tables

**Table 1:** Baseline characteristics of subjects.

Variables	Placebo (*n* = 29)	Probiotics (*n* = 30)	*p*-Value
Gender (*n*, female/male)	8/21	5/25	0.486
Smoker (Y/N)	9/20	7/23	0.710
Drinker (Y/N)	21/8	25/5	0.486
Age (years)	39.34 ± 1.61	35.7 ± 1.44	0.096
Height (m)	171.62 ± 1.69	171.32 ± 1.34	0.891
Weight (kg)	79.07 ± 1.85	79.21 ± 2.19	0.961
BMI (kg/m^2^)	26.81 ± 0.47	26.87 ± 0.52	0.880
Waist circumference (cm)	94.92 ± 1.32	93.99 ± 1.88	0.217
Hip circumference (cm)	101.97 ± 0.83	101.58 ± 0.93	0.752
Percent body fat (%)	30.17 ± 1.22	27.53 ± 1.34	0.151
Body fat mass (kg)	23.66 ± 0.95	21.86 ± 1.3	0.268
Lean body mass (kg)	55.41 ± 1.78	57.35 ± 1.77	0.443
Visceral fat area (cm^2^)	107.98 ± 5.49	97 ± 6.79	0.085
SBP (mmHg)	135.93 ± 1.66	132.47 ± 2.16	0.210
DBP (mmHg)	132.66 ± 2.09	131.27 ± 2.28	0.656
HR (beats/min)	83.41 ± 1.98	80.1 ± 1.61	0.079
Total cholesterol (mg/dL)	200 ± 5.28	215.93 ± 6.47	0.161
HDL-cholesterol (mg/dL)	53.45 ± 2.15	52.5 ± 2.59	0.462
LDL-cholesterol (mg/dL)	132.31 ± 6.79	148.8 ± 6.47	0.084
Triglyceride (mg/dL)	137.34 ± 11.02	140.87 ± 16.48	0.688
Insulin (μIU/mL)	7.08 ± 0.83	6.16 ± 0.6	0.400
Leptin (ng/mL)	19.13 ± 12.38	15.31 ± 2.08	0.084
Adiponectin (ng/mL)	2741.52 ± 306.7	2222.47 ± 214.29	0.240
hs-CRP (mg/dL)	0.99 ± 0.28	1.62 ± 0.59	0.103

Result are means ± SEM. A chi-square test was performed on categorical variables. An independent *t*-test was performed on continuous variables.

**Table 2:** Anthropometric measurements, body composition, and visceral fat area.

Variables	Placebo (*n* = 29)			Probiotics (*n* = 30)			*p*-Value
	Baseline	12 Weeks	Change	Baseline	12 Weeks	Change	
Body weight (kg)	79.07 ± 1.85	79.99 ± 1.95	0.93 ± 0.28	79.21 ± 2.19	78.74 ± 2.12	−0.47 ± 0.29	0.001 ^T^
BMI (kg/m^2^)	26.81 ± 0.47	27.13 ± 0.53	0.32 ± 0.1	26.87 ± 0.52	26.73 ± 0.51	−0.15 ± 0.1	<0.001 ^T^
Waist circumference (cm)	94.92 ± 1.32	96.23 ± 1.52	1.31 ± 0.5	93.99 ± 1.88	93.58 ± 1.93	−0.41 ± 0.37	0.007 ^T^
Hip circumference (cm)	101.97 ± 0.83	101.59 ± 0.89	−0.38 ± 0.44	101.58 ± 1.93	101.08 ± 0.89	−0.5 ± 0.21	0.062 ^M^
Percent body fat (%)	30.17 ± 1.22	30.24 ± 1.3	0.08 ± 0.2	27.53 ± 1.34	27.29 ± 1.35	−0.23 ± 0.28	0.366 ^T^
Body fat mass (kg)	23.66 ± 0.95	24.05 ± 1.11	0.39 ± 0.22	21.86 ± 1.3	21.58 ± 1.31	−0.28 ± 0.24	0.043 ^T^
Lean body mass (kg)	55.41 ± 1.78	55.92 ± 1.81	0.51 ± 0.2	57.35 ± 1.77	57.16 ± 1.72	−0.18 ± 0.25	0.032 ^T^
Visceral fat area (cm^2^)	107.98 ± 5.49	110.81 ± 6.1	2.82 ± 1.33	97 ± 6.8	95.34 ± 7	−1.67 ± 1.44	0.025 ^T^

The data points correspond to the mean ± SEM. Differences in changes in the mean values from week 0 to week 12 between placebo and probiotics groups. *p*-value obtained from independent *t*-test ^(T)^ or Mann–Whitney U Test ^(M)^.

**Table 3:** Biochemical measurements.

Variables	Placebo (*n* = 29)			Probiotics (*n* = 30)			*p*-Value
	Baseline	12 Weeks	Change	Baseline	12 Weeks	Change	
Total cholesterol (mg/dL)	200 ± 5.28	203.69 ± 6.07	3.69 ± 3.83	215.93 ± 6.47	215.33 ± 7.81	−0.6 ± 5.04	0.501 ^M^
HDL-cholesterol (mg/dL)	53.45 ± 2.15	52 ± 1.85	−1.45 ± 1.15	52.5 ± 2.59	52.1 ± 2.93	−0.4 ± 1.18	0.527 ^T^
LDL-cholesterol (mg/dL)	132.31 ± 6.79	146.1 ± 7.13	13.79 ± 4.14	148.8 ± 6.47	153.3 ± 8.03	4.5 ± 4.59	0.139 ^T^
Triglyceride (mg/dL)	137.34 ± 11.02	145.59 ± 12.25	8.24 ± 9.99	140.87 ± 16.48	157.93 ± 17.66	17.07 ± 11.4	0.563 ^M^
Insulin (μIU/mL)	7.08 ± 0.83	6.32 ± 0.52	−0.77 ± 0.79	6.16 ± 0.6	8.9 ± 3.66	2.74 ± 3.55	0.934 ^M^
Leptin (ng/mL)	19.13 ± 2.38	21.83 ± 2.7	2.7 ± 1.59	15.31 ± 20.8	14.55 ± 1.82	−0.76 ± 0.88	0.117 ^M^
Adiponectin (ng/mL)	2741.52 ± 306.7	2494.69 ± 265.53	−246.83 ± 178.68	2222.47 ± 214.29	2355.8 ± 190.75	133.33 ± 150.47	0.046 ^M^
hs-CRP	0.99 ± 0.28	0.78 ± 0.18	−0.21 ± 0.2	1.62 ± 0.59	4.74 ± 3.96	3.13 ± 3.63	0.619 ^M^

The data points correspond to the mean ± SEM. Differences in changes in mean values from week 0 to week 12 between placebo and probiotics groups. *p*-value obtained from independent *t*-test ^(T)^ or Mann–Whitney U Test ^(M)^.

## Data Availability

All datasets have been deposited in NCBI Gene Expression Omnibus with the accession code GSE202489.

## Supplementary Materials

The following supporting information can be downloaded at: https://www.mdpi.com/article/10.3390/nu14122484/s1, Figure S1: Acid and bile tolerance of *Lactobacillus* strains.
