# Supplementary material for: Effects of Lactobacillus curvatus HY7601 and Lactobacillus plantarum KY1032 on Overweight and the Gut Microbiota in Humans: Randomized, Double-Blinded, Placebo-Controlled Clinical Trial
Source: Nutrients. 2022 Jun 15;14(12):2484. doi: 10.3390/nu14122484 (PMC9228474; doi:10.3390/nu14122484)
Supplement: Supplementary file 1 [file nutrients-14-02484-s001.zip › nutrients-1730499-supplementary.pdf]

nutrients

MDPI

Supplement data

Effects of *Lactobacillus curvatus* HY7601 and *Lactobacillus plantarum* KY1032 on Overweight and the Gut Microbiota in Humans: Randomized, Double-Blinded, Placebo-Controlled Clinical Trial

Sung-Joon Mo [1,†], Kippeum Lee [1,†], Hyoung-Ju Hong [2], Dong-Ki Hong [1], Seung-Hee Jung [1], Soo-Dong Park [1], Jae-Jung Shim [1] and Jung-Lyoul Lee [1,*]

[1] R&BD Center, Hy Co., Ltd., 22, Giheungdanji-ro 24beon-gil, Giheung-gu, Yongin-si 17086, Korea; stal6000@hy.co.kr (S.-J.M.); joy4917@hanmail.net (K.L.); dkhong@hy.co.kr (D.-K.H.); jungsh@hy.co.kr (S.-H.J.); soodpark@hy.co.kr (S.-D.P.); jjshim@hy.co.kr (J.-J.S.)

[2] Department of Gastroenterology, Vievis Namuh Hospital, 627, Nonhyeon-ro, Gangnam-gu, Seoul 06117, Korea; h2forever@vievisnamuh.com

* Correspondence: jlleesk@hy.co.kr; Tel.: +82-70-7835-6001

† These authors contributed equally to this work.

Citation: Mo, S.-J.; Lee, K.; Hong, H.-J.; Hong, D.-K.; Jung, S.-H.; Park, S.-D.; Shim, J.-J.; Lee, J.-L. Effects of *Lactobacillus curvatus* HY7601 and *Lactobacillus plantarum* KY1032 on Overweight and the Gut Microbiota in Humans: Randomized, Double-Blinded, Placebo-Controlled Clinical Trial. *Nutrients* **2022**, *14*, 2484. https://doi.org/10.3390/nu14122484

Academic Editor: Christopher Papandreou

Received: 1 May 2022
Accepted: 14 June 2022
Published: 15 June 2022

Publisher's Note: MDPI stays neutral with regard to jurisdictional claims in published maps and institutional affiliations.

Copyright: © 2022 by the authors. Submitted for possible open access publication under the terms and conditions of the Creative Commons Attribution (CC BY) license (https://creativecommons.org/licenses/by/4.0/).

1. Material and Method

1.1. Acid and bile tolerance Acid and bile tolerance

Acid and bile tolerance of *Lactobacillus* strains were evaluated, as described by Chung et al. [1]. Briefly, 100 µL of overnight bacterial cultures in MRS broth were inoculated into 10 mL sterile phosphate buffered saline (PBS). Acid resistance experiments were performed by incubation at 37 °C for 2 h with the addition of 1% pepsin in saline with a pH value of 2.5 (10M HCl, adjusted using Merck). Bile tolerance experiments were performed by incubation at 37 °C for 2 h in saline with a pH value of 8.0 (10 M HCl, adjusted using Merck) supplemented with 0.3% bile. In each experiment, the *Lactobacillus* strains incubated for 2 hours at 37°C in PBS with a pH of 6.2 was set as a control group. Counting of strain populations was performed by surface plating on MRS agar followed by incubation at 37 °C for 24 h. The survival rate was calculated as follows.

Percent survival = final (cfu/mL)/control (cfu/mL) × 100.

1.2. Resistance to the Effects of the GI Tract

We evaluated *Lactobacillus* strains by measuring survival rate in physiological conditions similar to those of the human GIT, as described by Jung et al. [2]. To this end, in vitro digestion tests were performed to evaluate resistance to saliva, gastric juice, and bile salts. Simulated Salivary Fluid (SSF), simulated gastric fluid (SGF), and simulated intestinal fluid (SIF) were preheated to 37 °C prior to in vitro digestion of bacteria. Then, 5 mL Lactobacillus strain culture was poured into a 50 mL conical tube. The oral simulation was performed by adding 26 µL of 0.3 M $CaCl_2$ solution and 4 mL of 6.55 mg/mL α-amylase solution in SSF. The pH was then adjusted to 7.0 with 1 M NaOH, and the mixture was incubated at 37 °C for 2 min. The gastric fluid simulation was performed by adding 6 µL of 0.3 mol/L $CaCl_2$, 694 µL distilled water, and 9.1 mL of 0.07 mg/mL pepsin to SGF. The pH was adjusted to 3 with 1 M HCl, and the mixture was incubated at 37 °C with continuous shaking for 2 h. The intestinal phase was simulated by adding 40 µL of 0.3 M $CaCl_2$, 1.31 mL distilled water, 2.5 mL of 160 mM bile extract, and 16 mL of 22.15 mg/mL pancreatic solution. The pH was raised to 7.0 by adding 1 M NaOH, and the mixture was incubated at 37 °C for 2 h with shaking. For each step, a control group cultured in PBS was set. At the end of each simulation, aliquots of Lactobacillus strains were collected, and viable

cell counts were measured to determine the viability after simulated saliva, gastric fluid, and intestinal fluid digestions.

2. Result

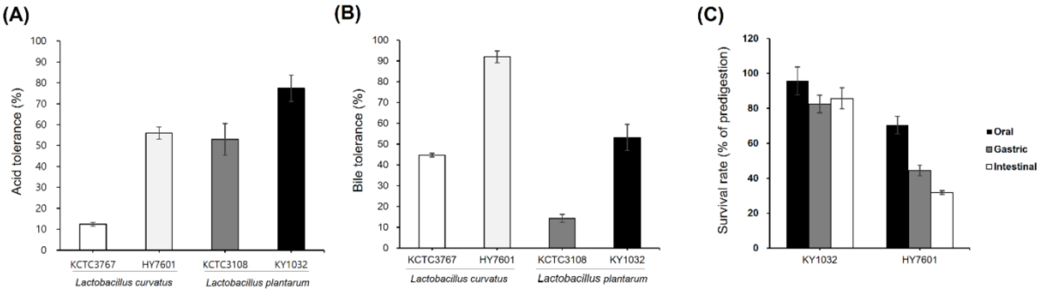

Figure S1. Acid and bile tolerance of *Lactobacillus* strains. (**A**) Acid tolerance of *Lactobacillus* strains in PBS with pH value 2.5 and addition 1% pepsin and (**B**) bile tolerance of *Lactobacillus* strains in PBS with pH value 8.0 and addition 0.3% bile. (**C**) The survival rate of KY1032 and HY7601 during simulated gastrointestinal tract. Data are represented as the mean ± standard deviation (SD) of three independent experiments. KCTC3767, *Lactobacillus curvatus* type strain; KCTC3108, *Lactobacillus plantarum* type strain.

References

1. Chung, H.; Kim, Y.; Chun, S.; Ji, G.E. Screening and selection of acid and bile resistant bifidobacteria. *International journal of food microbiology* **1999**, *47*, 25-32.
2. Jung, S.H.; Hong, D.K.; Bang, S.-J.; Heo, K.; Sim, J.-J.; Lee, J.-L. The functional properties of Lactobacillus casei hy2782 are affected by the fermentation time. *Applied Sciences* **2021**, *11*, 2481.
